# Influence of PER-7 on cefiderocol susceptibility in clinical isolates of *Acinetobacter baumannii* producing OXA-23 or OXA-72 carbapenemases

**DOI:** 10.1093/jacamr/dlaf188

**Published:** 2025-10-18

**Authors:** Pia Turowski, Martina Cremanns, Jessica Eisfeld, Sören Gatermann, Niels Pfennigwerth

**Affiliations:** German National Reference Centre for Multidrug-Resistant Gram-Negative Bacteria, Department of Medical Microbiology, Ruhr-University Bochum, Bochum, Germany; Department of Environmental Microbiology, North Rhine-Westphalia State Agency for Nature, Environment and Consumer Protection, Recklinghausen, Germany; German National Reference Centre for Multidrug-Resistant Gram-Negative Bacteria, Department of Medical Microbiology, Ruhr-University Bochum, Bochum, Germany; German National Reference Centre for Multidrug-Resistant Gram-Negative Bacteria, Department of Medical Microbiology, Ruhr-University Bochum, Bochum, Germany; German National Reference Centre for Multidrug-Resistant Gram-Negative Bacteria, Department of Medical Microbiology, Ruhr-University Bochum, Bochum, Germany

*Acinetobacter baumannii* poses a significant challenge in clinical settings due to its ability to cause nosocomial infections and its extensive resistance to multiple antibiotics. Between March 2022 and September 2023, 759 out of 983 (77%) isolates of *A. baumannii* submitted to the German National Reference Centre for Multidrug-resistant Gram-negative Bacteria produced an OXA-23 and/or OXA-72 carbapenemase.^1,2^ These infections are difficult to treat, as only a few effective antibiotics remain. One potential therapeutic option is cefiderocol, a catechol-substituted siderophore cephalosporin. However, in the mentioned time period, 181 (24%) isolates showed inhibition zone diameters <17 mm for cefiderocol, corresponding to the pharmacokinetic/pharmacodynamic (PK/PD) EUCAST breakpoint of >2 mg/L.^3^ We analysed 39 randomly selected OXA-23 (*n* = 31) or OXA-72 (*n* = 8) producing cefiderocol-resistant *A. baumannii* strains by PCR and/or whole-genome sequencing. Given that OXA-23 and OXA-72 are found in the vast majority of carbapenem-resistant *A. baumannii* in Germany, we specifically focused on cefiderocol-resistant isolates carrying these enzymes to ensure clinical and epidemiological relevance. Whole-genome sequencing revealed no mutations in previously identified resistance determinants such as PBP3, *pirA*, or *piuA*, and no NDM-type or other metallo-β-lactamases were detected, thereby excluding their involvement in the reduced cefiderocol susceptibility of these strains. All isolates harboured the extended-spectrum β-lactamase PER-7. PER-7 shows hydrolytic activity against broad-spectrum cephalosporins, aztreonam, and, to a limited extent, carbapenems.^4^ Previous studies by Poirel *et al.* reported cefiderocol-resistant *A. baumannii* isolates producing OXA-23 and PER-7, implicating a role for PER-7 in cefiderocol resistance.^5^ Heterologous expression in *Escherichia coli* TOP10 and *Pseudomonas aeruginosa* PAO1 raised cefiderocol MICs from ≤0.125 to 4 mg/L in *E. coli*, and from 0.5 to 8 mg/L in *P. aeruginosa*.^6^ However, *bla*_PER-7_ was not cloned into *A. baumannii* in the respective study, and the exact influence on cefiderocol resistance in this species remains not fully known.

Consequently, the aim of this study was to investigate the effect of PER-7 production on cefiderocol susceptibility in *A. baumannii*. Therefore, the *bla*_PER-7_ gene was amplified from *A. baumannii* NRZ-88408 and ligated into the expression plasmid pVRL-1 (BCCM, https://bccm.belspo.be) after XhoI and PstI digestion. The recombinant plasmid was transformed into *E. coli* DH5α, *A. baumannii* ATCC 17978, and four randomly selected, non-duplicate clinical *A. baumannii* isolates (NRZ-89738, NRZ-90235, NRZ-90408, NRZ-92586) producing OXA-23 or OXA-72, all with cefiderocol inhibition zone diameters ≥17 mm. Cefiderocol susceptibility was tested in triplicate by manual broth microdilution in iron-depleted, cation-adjusted Mueller-Hinton broth (concentration range: 0.03–32 mg/L).^7^

Heterologous expression of PER-7 led to increased MICs for cephalosporins and aztreonam in all strains, and a significant rise in carbapenem MICs in *A. baumannii* ATCC 17978, where MICs of imipenem and meropenem increased from 0.125 to 4 and 0.5 mg/L, respectively (Table 1). Similarly, the MIC for ertapenem increased from 1 to 4 mg/L. Despite these increases, MICs for meropenem and imipenem remained within the susceptible or susceptible, increased exposure range, indicating that *bla*_PER-7_ alone does not confer full resistance. In contrast, *E. coli* DH5α showed no change in carbapenem MICs. In the four clinical *A. baumannii* isolates, carbapenem resistance was already conferred by oxacillinases OXA-23 or OXA-72.^8^ PER-7 expression led to an increase in cefiderocol MICs in *E. coli* and all tested *A. baumannii* isolates (Table 1). MIC values rose to > 32 mg/L in *A. baumannii*, surpassing clinically relevant PK/PD thresholds (>2 mg/L) as defined by EUCAST.^3^ The increases in cefiderocol MICs observed in our *in vitro* models were consistent with resistance levels detected in the clinical PER-7 isolates. MICs for ceftazidime-avibactam and aztreonam-avibactam suggested that PER-7 is not fully inhibited by avibactam (Table 1). In *E. coli* DH5α, production of PER-7 led to a 1024-fold MIC increase for aztreonam (up to >64 mg/L), while MICs for aztreonam-avibactam increased only 62-fold, to 2 mg/L, indicating an incomplete inhibition. This is in line with the findings of Poirel *et al.,* who showed that *E. coli* TOP10 with and without production of PER-7 showed aztreonam and aztreonam-avibactam MICs of >256 and 16 mg/L, respectively, which also indicates an incomplete inhibition. In *A. baumannii*, no inhibitory effect of avibactam was observed; however, this was expected, as both ceftazidime-avibactam and aztreonam-avibactam are no adequate drugs against *A. baumannii* due to intrinsic resistance mechanisms, such as the hydrolysis of ceftazidime and aztreonam by the chromosomally encoded ADC β-lactamase, and the inability of avibactam to cross the *Acinetobacter* outer membrane.^9^ This likely masked the effect of PER-7 inhibition. As cefiderocol was not tested in combination with avibactam in our study, any potential synergistic effects could not be evaluated.

**Table 1.** MIC (mg/L) of *E. coli* DH5α, *A. baumannii* ATCC 17 978 and OXA-23 (NRZ-89738, NRZ-90408, NRZ-92586) or OXA-72 (NRZ-90235) producing *A. baumannii* with or without production of PER-7

Species and antimicrobial agent	*Escherichia coli*		*Acinetobacter baumannii*									
	DH5α		ATCC 17 978		NRZ-89738 (OXA-23)		NRZ-90408 (OXA-23)		NRZ-92586 (OXA-23)		NRZ-90235 (OXA-72)	
	− PER-7	+ PER-7	− PER-7	+ PER-7	− PER-7	+ PER-7	− PER-7	+ PER-7	− PER-7	+ PER-7	− PER-7	+ PER-7
Ceftazidime-Avibactam	≤ 1	8	16	> 32	32	> 32	32	> 32	> 32	> 32	16	> 32
Ceftolozane-Tazobactam	≤ 0.25	> 8	≤ 0.25	> 8	> 8	> 8	8	> 8	> 8	> 8	> 8	> 8
Cefiderocol	0.125	2	0.25	> 32	1	> 32	0.5	> 32	1	> 32	1	> 32
Imipenem	0.25	0.25	0.125	4	64	32	16	16	64	64	64	64
Meropenem	≤ 0.03	≤ 0.03	0.125	0.5	> 64	> 64	32	32	64	32	> 64	> 64
Ertapenem	≤ 0.06	≤ 0.06	1	4	> 64	> 64	> 64	> 64	> 64	64	> 64	> 64
Imipenem-Relebactam	≤ 0.25	≤ 0.25	≤ 0.25	0.5	> 8	> 8	> 8	> 8	> 8	> 8	> 8	> 8
Meropenem-Vaborbactam	≤ 1	≤ 1	≤ 1	≤ 1	> 32	> 32	32	32	32	32	> 32	> 32
Aztreonam	0.06	> 64	16	> 64	16	> 64	32	> 64	64	> 64	16	> 64
Aztreonam-Avibactam	≤ 0.03	2	16	> 64	16	> 64	32	> 64	32	> 64	16	> 64
Colistin	1	1	2	2	2	1	1	2	2	2	2	2
Tigecycline	≤ 0.125	≤ 0.125	≤ 0.125	≤ 0.125	0.5	0.5	0.25	≤ 0.125	0.5	0.5	0.5	0.5

In conclusion, our study confirms that the presence of PER-7 contributes to cefiderocol resistance in *A. baumannii*. The frequent localization of *bla*_PER-7_ on plasmids facilitates its rapid dissemination and transfer to other bacteria.^10^ These findings highlight the limited therapeutic options for infections caused by cefiderocol-resistant *A. baumannii*. To date, there is insufficient evidence to suggest that cefiderocol is an effective treatment option for such isolates.
